# Depressive and Other Adverse CNS Effects of Fluoroquinolones

**DOI:** 10.3390/ph16081105

**Published:** 2023-08-04

**Authors:** Piotr Wierzbiński, Joanna Hubska, Michał Henzler, Bartłomiej Kucharski, Rafał Bieś, Marek Krzystanek

**Affiliations:** 1 Psychiatry Outpatient Clinic, Okoniowa 5/33, 91-498 Łódź, Poland; 2 Faculty of Medicine, Medical University of Warsaw, Żwirki i Wigury 61, 02-091 Warsaw, Poland; s073907@student.wum.edu.pl (J.H.); s073759@student.wum.edu.pl (M.H.); s075177@student.wum.edu.pl (B.K.); 3 Medical Students’ Association, Department and Clinic of Psychiatric Rehabilitation, Faculty of Medical Sciences, Medical University of Silesia in Katowice, Ziołowa 45/47, 40-635 Katowice, Poland; s82700@365.sum.edu.pl; 4 Department and Clinic of Psychiatric Rehabilitation, Faculty of Medical Sciences, Medical University of Silesia in Katowice, Ziołowa 45/47, 40-635 Katowice, Poland; m.krzystanek@sum.edu.pl

**Keywords:** fluoroquinolones, side effects, adverse effects, depression, anxiety, psychotic symptoms, disturbances of consciousness

## Abstract

Fluoroquinolones (FQs) are widely used drugs around the world. This is a result of their broad spectrum of antibacterial activity, high bioavailability, and known efficacy. Since they appeared on the market, their prescribing frequency has gradually increased. In 2011, FQs became the third most prescribed class of antibiotics in the US. Widespread use of these drugs resulted in an increasing number of reported side effects. In 2016, the FDA warned about significant side effects, including mental disorders in the form of anxiety, psychotic symptoms, insomnia, and depression. Psychiatric adverse reactions to FQs occur with a frequency of 1 to 4.4% and the mechanism of their formation is not entirely clear. It is believed that the antagonistic effect of FQs on the GABA receptor or interaction with the main receptor for the glutamatergic system—NMDA—is responsible for this. The paper is a structured review of 68 selected publications and the latest summary of CNS adverse effects that occur during FQ use. Prescribers should be aware of the risk factors for FQ toxicity, including elderly patients with underlying medical conditions or receiving concomitant medication; however, these adverse events may also occur in other groups of patients.

## 1. Introduction

Fluoroquinolones (FQs) are antibiotics commonly used worldwide due to their broad spectrum of antibacterial activity, high bioavailability, and efficacy. They have predominantly been used to treat bacterial infections caused by both Gram-positive and Gram-negative bacteria, including, but not limited to, urinary tract infections, pneumonia, sinusitis, tuberculosis, and sexually transmitted infections [1]. This class of oral antibiotics with convenient dosing schedules and high bioavailability was readily accepted and has been commonly prescribed at increasing rates since the late 1980s, when they became available [1,2]. In 2011, FQs were the third most prescribed antibiotic class in the United States (US) [3]. The widespread use of FQs has led to an increase in reports of adverse drug reactions (ADRs) to them, making it possible to precisely describe them. Consequently, in 2008, the FDA added a boxed warning regarding the risk of tendinitis and tendon rupture with the FQ class [4]; in 2011, a boxed warning regarding worsening myasthenia gravis symptoms was added [4]; and in 2013, the drug class warnings were updated to include irreversible peripheral neuropathy [4]. In 2016, the FDA revised the boxed warning for all oral and injectable FQs due to the risks of serious side effects, including CNS ADRs such as psychosis, anxiety, insomnia, depression, hallucinations, suicidal thoughts, and confusion [5,6]. The most recent warnings, issued in 2018, consisting of concern for CNS ADRs, risk of hypoglycemia, and increased aortic rupture or tear were added to the FQ labeling [4]. The combination of these warnings and collateral damage has led to some FQs being taken off market and removed from clinical use [7]. Interestingly, none of the FQs were withdrawn from the market because of CNS ADRs [7]. Notably, the FDA’s warning about psychiatric ADRs was issued relatively late compared to the other warnings. These facts may reflect the under-recognition and under-reporting of CNS ADRs of FQs by both patients and physicians. 

Fluoroquinolone ADRs related to the central nervous system (CNS) are the second most frequent after gastrointestinal-related ADRs. Neuropsychiatric ADRs are estimated to occur in 1–4.4% patients and range from mild (confusion, irritability, and insomnia) to severe (encephalopathy, seizures, suicidal depression, catatonia, psychosis, and mania) [8]. The underlying mechanisms are not clear, although it is postulated that they act as low-affinity gamma-aminobutyric acid (GABA) antagonists [7]; other researchers postulate that interactions with the N-methyl-D-aspartate (NMDA) receptor might be responsible [8]. 

According to the statistics, in 2014, approximately 22 million unique patients received a dispensed prescription for a selected oral FQ from US outpatient retail pharmacies [6]. In 2016, FQs ranked fourth among the most frequently prescribed antibiotics in the US [9]. The misuse and overuse of FQs have contributed to the development and spread of resistance. Inappropriate prescribing practices, including using FQs for non-bacterial infections or as a first-line therapy when other antibiotics would be more appropriate, have accelerated resistance. The development of antimicrobial resistance to FQs can occur through various mechanisms. One common mechanism is the alteration of the target site of the antibiotic. Fluoroquinolones work by inhibiting enzymes called DNA gyrase and topoisomerase IV, which are essential for bacterial DNA replication. Mutations in the genes encoding these enzymes can lead to structural changes that reduce the binding affinity of FQs, rendering them less effective against bacteria. Another mechanism of resistance is the active efflux of the antibiotic from the bacterial cell. Furthermore, bacteria can acquire resistance genes through horizontal gene transfer, which causes resistance to FQs to spread rapidly within bacterial populations. Antimicrobial resistance to FQs poses significant challenges in the treatment of bacterial infections as it limits the available treatment options and can lead to treatment failure, prolonged illness, and increased healthcare costs.

Nowadays, due to increasing antimicrobial resistance and growing awareness of the harmful side effects, the frequency of prescribing FQs is declining. In the US, from 2015 to 2019, FQ prescription fills decreased annually by 10.8% [10]. However, FQs still rank high among the most used antibiotics, both in Europe and the US. Notably, some FQs were placed on the World Health Organization Model List of Essential Medicines—22nd List, 2021, and, for example, ciprofloxacin is indicated as the first-choice antibiotic for acute invasive bacterial diarrhea/dysentery, enteric fever, low-risk febrile neutropenia, pyelonephritis, and prostatitis (mild to moderate) [11].

While the somatic ADRs of FQs have been extensively researched and widely described, CNS ADRs have received less attention. The etiology and pathogenesis of psychiatric ADRs remain poorly understood. The importance of such events is significant, as the misdiagnosis of a CNS ADR entails great suffering for the patient affected as well as unnecessary costs for the healthcare system. To date, it has not been determined what characteristics of patients may increase the risk of psychiatric ADRs from FQs. Moreover, there are no guidelines on how these conditions should be treated. Although there are several hypotheses about the FQ mechanisms underlying CNS symptoms, none have been officially confirmed so far. 

Our objective in this review was to offer an approach to identifying and managing CNS ADRs from FQs based on the characteristics of symptoms and clinical features of affected patients. In recent years, there have been attempts to study the effects of quinolones on the CNS more thoroughly and two relevant reviews were published [7,12], upon which we hope to elaborate and expand.

## 2. Materials and Methods

### 2.1. Search Strategy

A comprehensive search of the literature published from inception to 20 December 2020 was conducted according to the PRISMA guidelines on the PubMed and Web of Science databases. The systematic review was registered in the PROSPERO registry (no CRD42023422578). The search terms included “ciprofloxacin”, “ofloxacin”, “gemifloxacin”, “levofloxacin”, “moxifloxacin”, “delafloxacin” and “mania”, “manic”, “psychosis”, “psychotic”, “delirium”, “depression”, “hallucinations”, “suiccidal attempt”, “dellusions”, “dystonia”, “psychiatric adverse drug reactions”. The search terms were combinations formed from each of the drugs along with each of the symptoms. The two investigators (PW and JH) independently screened all articles for those that met the broad inclusion criteria. Additional studies were identified through a physical search of bibliographies from primary articles.

### 2.2. Eligible Studies

**Figure 1:**
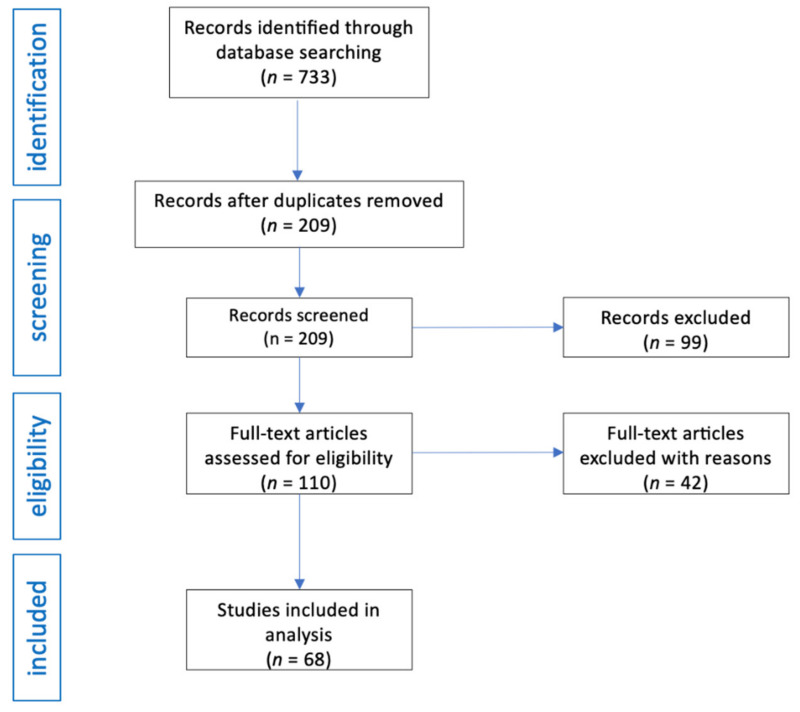
Flow diagram of studies analysis and selection for review.

A total of 733 abstracts were identified in the initial search. After removal of duplicates, 209 studies were further evaluated for key terms and relevance to the aims of the review. After title and abstract screening, 99 articles were removed due to not meeting the inclusion criteria. Of the 110 abstracts identified, a further 37 articles were removed after full text screening and another 5 articles were excluded during data extraction. Finally, a total of 68 publications, with 73 cases described, were included in this review. All the articles included were case reports. The flow diagram of the analysis is presented in Figure 1.

### 2.3. Inclusion and Exclusion Criteria

Articles referring to FQs and psychiatric disorders were included in which the psychiatric disorders were mainly associated with the use of an FQ [13]. Cases in which patients had been taking medications significantly affecting the CNS (opioids, benzodiazepines, 5-hydroxytryptamine antagonists, dopamine agonists, and theophylline) or had a history of primary psychotic disorders or a history of substance abuse were also included, as all these cases described a direct association of FQ intake with the onset of previously absent psychiatric (CNS) symptoms. The population of interest was patients of all ages. One relevant study with a large sample was found but was not considered because it included delirium with psychotic symptoms in the same group. Regarding language, both English and non-English languages were included. Non-English publications were translated using online dictionaries.

### 2.4. Data Extraction

Citations and abstracts of the search results were stored in Endnote X9 for evaluation and selection. The following data were extracted: type of study, number of cases, sex, age, co-morbidities, past medical history (including CNS disorders and substance abuse history), FQ involved, dose, route of administration, and psychiatric symptoms (indication for prescription, time of the onset and resolution, description, and management).

### 2.5. Data Quality

To assess the risk of bias and study quality in quantitative studies, the Effective Public Health Practice Project’s (EPHPP) Quality Assessment Tool for Quantitative Studies (QATQS) was used (Thomas et al. (2004); Armijo et al. (2012)). This tool enables quality evaluation of a wide range of study designs, including RCTs, observational studies with and without control groups, and case studies. The instrument contains eight different sections, each with multiple questions: selection bias, study design, confounders, blinding, data collection methods, withdrawals and drop-outs, intervention integrity, and analyses. Each section receives a score of 1 (strong), 2 (moderate), or 3 (weak), and a final score is determined by the number of ‘weak’ ratings. A strong rating is given to a study if there is no weak component score. A moderate rating is given with one weak component score. A weak rating is given with two or more component rating scores.

## 3. Results

### 3.1. Fluoroquinolone

Among the 68 eligible articles, the highest incidence of CNS ADRs was reported with levofloxacin (27 cases) and ciprofloxacin (30 cases). Ofloxacin was used in eight cases, moxifloxacin in six cases, and gemifloxacin in two cases. Delafloxacin was not mentioned in any of the eligible publications (Table 1). 

An extended description of the cases extracted from the analyzed literature is presented in Table S1 in the Supplementary Materials. 

### 3.2. Age and Sex 

Of the 72 patients described in the reports, 42 were women and 30 were men. The mean age was 45,14 years and the median was 45, covering cases of patients aged 2 [25] to 85 [49] years. Only 1onecase did not report the specific age [27]. Interestingly, there there were five cases in which the patients were children aged 2 [25], 4 [68], 5 [69], 13 [30], and 17 [26] years old.

### 3.3. Indications for Fluoroquinolone Therapy 

The most frequent reasons for prescribing fluoroquinolone treatment were a respiratory tract infection (a total of 29 cases), a urinary tract infection (17 cases), and gastrointestinal tract infections (a total of 11 cases). Other frequent reasons for FQ administration were bacteriemia [24,43,61,74,78] and perioperative prophylaxis [25,35,43,56,71,81]. In a small number of cases, FQs were used for the treatment of prostatitis [50], pelvic inflammatory disease [72], tuberculosis [57], osteoarticular infections [49], sinusitis [17,38], conjunctivitis [58], meningitidis [64], abscesses [16,51], and ulcers [37]. In two cases, FQs were introduced as an empirical treatment [27,66].

### 3.4. Route of Administration and Dose

In the majority of cases, FQs were administered by oral (36/73 cases) and intravenous routes (18/73 cases). In one case, ciprofloxacin was administered in the form of eye drops and psychiatric symptoms occurred after the third dose [58]. No publications were found that reported other routes of administration. In most cases, FQs were assigned in therapeutic doses (63/73). Note a case of a 4-year-old girl, who was given quadruple the prescribed dose of ofloxacin due to a mistake (she was given the whole tablet instead of a quarter) [68]. Information about the route of FQ administration was missing in 22 cases, while information about the dose was missing in 9 cases.

### 3.5. Time of the Onset, Management, and Resolution of Psychiatric Symptoms

The time from the administration of FQ to the appearance of CNS symptoms was highly variable; it ranged from 10 min [26] to 3 weeks [50]. In 25 cases, symptoms appeared within 24 h, and in one case after more than 7 days [50].

In the majority of cases (52/73), CNS symptoms completely resolved within 72 h of FQ withdrawal. In only seven cases, the recovery time lasted a week or longer. The predominant treatment was withdrawal of FQs, which resulted in resolution of CNS symptoms in all cases. Antipsychotic treatment was included in 21 cases, most commonly with lorazepam (8 cases) or haloperidol (7 cases). 

### 3.6. Concomitant Medication

Fluoroquinolone treatment with concomitant antibiotics was documented in 30 of the 73 cases included. Metronidazole (12/30) and vancomycin (7/30) were the most frequent concomitant antibiotics used. Other medications simultaneously administered were paracetamol (6/71) [17,19,27,29,73,75] and non-steroidal anti-inflammatory drugs (NSAIDs) (4/71) [14,40,62,73]. In 11 cases, patients were treated with psychoactive drugs, including opioids [20,25,27,51,64,81], antipsychotics [31,81], benzodiazepines [25,27,64], 5-hydroxytryptamine antagonists [25,68,69], antiepileptic drugs [31,69], dopamine agonists [66], and antidepressants [31,72]. Theophylline was used in one case [32]. 

### 3.7. Comorbidities and Substance Abuse

The most common concomitant diseases were hypertension (14/73) and renal failure (8/73). Some cases of autoimmune diseases were noted, including multiple sclerosis [24,31], Sjogren’s syndrome [20,24], rheumatoid arthritis [20], ulcerative colitis [56], primary sclerosing cholangitis [56], and lupus erythematosus [74]. A past medical history of psychiatric disorders, including schizoaffective disorder [31], depression [50,51,55,72], suicidal behavior [50], and psychosis [20], was found. Parkinson’s disease was noted in one case [66]. Importantly, in some cases, there was suspicion that the FQ-induced psychotic episode could be the first episode of a primary psychotic disorder [47]. Note one case of a patient with a history of recurrent depression with suicidal thoughts, with each episode preceded by one-week treatment with ciprofloxacin for prostatitis [50].

A few cases of substance abuse, including alcohol [40], tobacco [14] and marijuana [44,78] smoking, and cocaine abuse [51], were noted.

### 3.8. CNS Adverse Drug Reactions from Fluoroquinolones

The most frequently reported diagnoses were delirium, found in 28 cases (5 cases of delirium accompanied by psychotic symptoms), and psychosis, which occurred in 27 cases. According to some authors, there is a high risk of error in differentiating delirium and psychosis [7,12]. 

The most frequent psychiatric symptoms were hallucinations (45/73): visual (28/45), auditory (15/45), and tactile (3/45). Thirty-three cases of psychomotor agitation, manifesting as irritability, restlessness, and emotional agitation, were observed. Sleep difficulties and/or insomnia were reported in 14 cases. Paranoid delusions were noticed in seven cases, while suicidal thoughts/attempts [23,35,42,50,52,70] were observed in six cases. Interestingly, one patient with suicidal thoughts reported that two of their family members also had psychiatric ADRs from ciprofloxacin [42]. Note a case of patient who attempted suicide while being on ciprofloxacin therapy and had received levofloxacin therapy a week earlier [52]. Notably, there was one case of a patient who reported recurrent episodes of depression, which were preceded by one-week therapy with ciprofloxacin each time [50].

## 4. Discussion

All classes of antimicrobials, including FQs, can trigger a variety of adverse CNS effects [82]. Of the FQs most frequently found in this review—ciprofloxacin and levofloxacin—it should be mentioned that, although it might suggest that they have a somewhat greater capacity to trigger CNS ADRs, it is more likely to be related to the frequency of prescription of these antibiotics. Levofloxacin, which appeared to be the main FQ involved, is considered as a drug with a low potential for neuropsychiatric ADRs, due to the lowest CNS penetration among FQs, and a low potential for interaction with the CYP, minimizing the risk of drug interactions [7]. However, some data suggest that a lower degree of CNS penetration does not always translate to a reduced rate of neurological ADRs [83]. Ciprofloxacin, which appears to be the second most common FQ involved in this review, has frequently been described as triggering CNS adverse effects, which also may be due to the high frequency of prescription. For example, Sellick, J. et al. (2018) indicated a prevalence of up to 3.7% of delirium/psychosis during ciprofloxacin therapy in older patients, which is more than estimated by post-marketing surveillance (<1%) [83]. Several psychiatric ADRs were also identified for ofloxacin, which according to some authors may be over-representative, as these drugs are used to a lesser extent than ciprofloxacin and levofloxacin [7,12]. 

Regarding age, most of the reported cases indicate patients in late adulthood. Older patients are more susceptible to developing CNS ADRs because of pharmacodynamic changes that occur with age, including alterations in volume of distribution, drug metabolism, and protein binding [7,84]. Furthermore, an advanced age usually correlates with numerous co-morbidities that can also increase the risk of psychiatric disorders. According to Velickovic-Radovanovic et al. (2019), renal failure, diabetes mellitus, neurological diseases, arteriosclerosis, acidosis, hypoxemia, and hyponatremia are conditions/diseases that increase the risk of neurotoxicity of FQs [15]. Thus, it seems that older patients absolutely require monitoring of their psychiatric condition during FQ therapy, especially if they have multiple concurrent diseases or are taking other medications.

In the majority of cases, FQs were administered by the oral or intravenous route. The intravenous route is generally preferred in severe infections, due to the higher bioavailability [12]. Therefore, CNS ADRs may be expected to occur more frequently after intravenous administration; however, no such correlation was observed for ciprofloxacin [85]. In this review, we found 6 cases of intravenous administration of ciprofloxacin, 10 cases of oral administration, and 10 cases with no information about the administration route; these outcomes do not allow to draw valid conclusions. Interestingly, Sellick, J. et al. (2018) showed an almost equivalent number of neuropsychiatric ADRs when FQs were administered orally and parenterally [83]. 

The dose of FQ seems to have a significant effect on the occurrence of CNS ADRs. It has been postulated that FQs affect the CNS through the competitive displacement of GABA from neuronal receptors, resulting in inhibition of GABAergic transmission. Importantly, this mechanism is dose dependent, directly relating to the antibiotic concentration at the receptor site [72]. Note the case described by Bhattacharya, A. et al., in which psychosis with hallucinations occurred after administration of a fourfold dose of ofloxacin [68]. The association between FQ over-dosage and psychiatric disorders was also recognized by Hall, C.E. et al., who described CNS ADRs in two females treated with ofloxacin for pelvic inflammatory disease [72]. In these cases, ofloxacin was administered in therapeutic doses, although still relatively high (400–800 mg/day) compared to doses for other indications, such as uncomplicated cystitis (200–400 mg/day) [72]. In contrast, Tripathi, A. et al. indicates that CNS ADRs can also occur with minimal doses of FQs, also because of ophthalmic administration [58]. 

According to the literature, co-administration of FQs and other medicines may also contribute to neuropsychiatric disorders. NSAIDs can potentiate the competitive inhibition of neuronal GABA receptors by quinolones which, consequently, may trigger the development of neurotoxicity [7,86]. NSAIDs are also associated with the decrease in renal blood flow due to the inhibition of prostaglandins; this may increase the quinolone concentration as renal elimination may be impaired [7]. Additionally, FQs with unsubstituted piperazinyl rings (e.g., ciprofloxacin) have a stronger interaction with NSAIDs [7,87,88]. The structure at position 7 of the quinolone affects the risk of NSAID-potentiated CNS events [87], while positions 1 and 7 influence the drug’s potency, pharmacokinetics, and potential for interaction with theophylline [89]. In the presence of a quinolone, the serum concentration of theophylline increases by about 20% and its clearance decreases by about 30% [7]. Moreover, neuropsychiatric ADRs, including seizures and visual hallucinations, have already been reported in patients using multiple drug therapy, including theophylline [90]. In conclusion, it seems that the dose of both NSAIDs and theophylline (but not limited to just the dose) should be reduced when quinolone therapy is started. 

The infections for which fluoroquinolones treatments were prescribed were diverse and involved most of the organ systems. The first most frequent cause was a respiratory tract infection, the second was a urinary tract infection, and the third was gastrointestinal infections. It should be noted that many of the mechanisms by which infections can cause or trigger neuropsychiatric symptoms are not fully understood, representing a combination of mechanisms in which inflammatory processes may play a pivotal role. Much attention is currently being paid to the gastrointestinal microbiota and its relationship with psychiatric disorders. It seems that a better understanding of gut–brain axis functioning may favor awareness of the ethology of many psychiatric disorders. Importantly, antibiotics in general affect the composition of the gut microbiota, and it can be speculated that this effect may be at least partially responsible for some of the psychiatric symptoms presented in this review.

In almost half of the cases, FQs were prescribed concomitantly with other antibiotics, most commonly with metronidazole. According to the literature, metronidazole is associated with CNS ADRs, including hallucinations, depression, confusion, dizziness, vertigo, and psychosis [64,91,92]. Nazef, C. et al. noticed the time coincidence of ciprofloxacin withdrawal and cessation of psychotic symptoms, while they did not observe such a property after metronidazole withdrawal [42]. Koul, S. et al. (2009) suggested that an idiosyncratic reaction between metronidazole and ofloxacin may be involved in the occurrence of psychosis [70]. Velickovic-Radovanovic, R. (2019) et al. postulated that psychotic disorders were caused by the potentiation of the neurotoxic effect of the respective drugs given simultaneously, i.e., levofloxacin and metronidazole [15].

The results from this review showed that most of the case reports were serious and associated with hospitalization or prolongation of the existing hospitalization [93]. The most frequent diagnoses were delirium and psychosis. Medication-induced delirium is a well-known entity, and antibiotics deserve special consideration as a common potential cause for delirium [14]. According to some authors, there is an extreme under-recognition and under-diagnosis of drug-induced delirium generally, and levofloxacin-induced delirium specifically [14]. Similarly, in the case of psychosis, the sparse literature indicates that FQ-induced psychosis is under recognized [20]. 

It is important to point out that in the majority of cases, psychiatric disorders completely resolve after discontinuation of FQ therapy. In one case, antipsychotics were started during ciprofloxacin therapy but did not result in resolution of delirium; the symptoms resolved only after ciprofloxacin was discontinued [81]. In another case, the patient was prescribed antipsychotic treatment with risperidone and citalopram; they finally did not take the prescribed drugs, but discontinued ofloxacin, and her psychotic symptoms resolved 4 days later [70]. Taking into consideration the positive outcomes of FQ withdrawal that was observed in the majority of cases, it seems that restraint in starting psychiatric treatment may be appropriate; moreover, the decision should be made by a psychiatrist aware of possible FQ contribution.

It seems that the pharmacokinetic properties of fluoroquinolones are not significant in the context of the appearance of CNS side effects. The authors believe that comorbidities, other drugs taken by patients, and the presence of inflammation are of great importance in the occurrence of psychiatric complications. Inflammation has a significant impact on the functioning of neurotransmitter systems in the brain, and the administration of an additional drug in this condition that will affect GABAergic and glutamatergic transmission disturbs the neurochemical balance in the brain. Therefore, the very fact of administering a fluoroquinolone, and not its pharmacokinetic properties, may be responsible for the occurrence of psychiatric complications. This probably requires further research.

This literature review has some limitations regarding how subjective the selection of articles was; we intentionally excluded articles about neurologic ADRs, as we aimed to characterize CNS ADRs only. Moreover, most of the case reports did not meet the causality criteria (e.g., Naranjo criteria [94]). Polypharmacy is one important issue which is very common in real clinical practice; thus, this issue could be further explored. In the current review, the discussion of this topic was limited by the often-limited detail of case reports available in medical databases. In the end, we included only case reports and case series; the only relevant observational study we initially wanted to include turned out to have an inadequate research methodology. It must not be forgotten that case reports are the bottom line of the evidence-based structure, which limits the scientific soundness of the review. Additionally, the poor quality of some case reports and the lack of detailed clinical information (e.g., cognitive condition of patients) might also introduce bias. In developing this systematic review, we followed earlier PRISMA guidelines [95]. The current version of the PRISMA guidelines is recommended for future developments on this topic. However, this review is comprehensive and attempts to systematically cover cases in which FQs induced CNS ADRs.

## 5. Conclusions

Consideration of antimicrobial side effects in differential diagnosis is vital, as the development of psychiatric symptoms after antimicrobial use can lead to incorrect diagnosis and treatment. Prescribers should be aware of the risk of FQ toxicity in everyone, especially in patients with multimorbidities or receiving concomitant medications. Multidisciplinary approaches are crucial to reveal the exact cause of FQ-induced adverse reactions. Appropriate patient education and prompt drug discontinuation in the event of an ADR are important considerations when prescribing FQs. Patients should remain vigilant to symptoms such as mood changes or hallucinations and report these promptly. Finally, further research is needed to confirm the underlying mechanisms of fluoroquinolone-associated psychiatric disorders.

## Figures and Tables

**Table 1:** Number of cases for each fluoroquinolone.

Fluoroquinolone	Number of Cases	Reference
Levofloxacin	30	[14,15,16,17,18,19,20,21,22,23,24,25,26,27,28,29,30,31,32,33,34,35,36,37,38,39,40]
Ciprofloxacin	27	[41,42,43,44,45,46,47,48,49,50,51,52,53,54,55,56,57,58,59,60,61,62,63,64,65,66]
Ofloxacin	8	[67,68,69,70,71,72]
Moxifloxacin	6	[73,74,75,76,77,78]
Gemifloxacin	2	[79,80]

## Data Availability

Data are contained within the article.

## Supplementary Materials

The following supporting information can be downloaded at: https://www.mdpi.com/article/10.3390/ph16081105/s1, Table S1: Studies on psychiatric side effects of fluoroquinolones. The risk of bias and study quality assessed with the Effective Public Health Practice Project’s Quality Assessment Tool for Quantitative Studies (QATQS) was presented as the global rating for each publication (3—weak).
