# Supplementary material for: Depressive and Other Adverse CNS Effects of Fluoroquinolones
Source: Pharmaceuticals (Basel). 2023 Aug 4;16(8):1105. doi: 10.3390/ph16081105 (PMC10459424; doi:10.3390/ph16081105)
Supplement: Supplementary file 1 [file pharmaceuticals-16-01105-s001.zip › pharmaceuticals-2470908-supplementary.pdf]

Table S1. Studies on psychiatric side effects of fluoroquinolones. The risk of bias and study quality assessed with the Effective Public Health Practice Project's Quality Assessment Tool for Quantitative Studies (QATQS) was presented as the global rating for each publication (3 – weak).

Authors and year	Type of study	N	Sex/age (years)	Dose	Administ ration route	Indications	Time of the onset of psychiatric symptoms	Concomitant medication	Psychiatric symptoms	Past medical history	History of CNS disorders and substance abuse	Time to the complete resolution of psychotic symptoms	Treatment of psychiatric symptoms	QATQS Global Rating
**Levofloxacin**														
Odeh, M., et al. [14]	CR	1	Man/76	500 mg/day	Per os	Acute bronchitis	3 days	Aspirin, ramipril, bisoprolol, Atorvastatin.	Delirium: impaired attention capacity, visual hallucinations, irritable, confused	Ischemic heart disease with stable angina pectoris, hypertension, hypercholesterolemia, mild renal failure, peripheral arterial disease	Heavy tobacco smoking of 15 years duration (stopped 16 years ago)	48 hours	Withdrawal	3
Velickovic-Radovanovic, R., et al. [15]	CR	1	Woman/48	500 mg/day	IV	Enterocolitis, urinary infection	3 days	Metronidazole, probiotic yeast culture *Saccharomyces boulardii,* carvedilol, ranitidine, calcium carbonate, B-complex vitamins	Psychotic disorder: confusion, disorientation, signs of delirium	Chronic kidney disease	No	4 days	Withdrawal, lorazepam, haloperidol	3
Muradian, M. and S. Khan et al. [16]	CR	1	Woman/23	ND	ND	Bilateral submandibul ar abscesses	3 days	Metronidazole, clindamycin	Psychosis: progressive paranoia and insomnia, visual hallucinations, flat affect, poor eye contact	Recurrent submandibular abscesses and a submental abscess, allergy to antibiotics including amoxicillin, ampicillin, and penicillin	No	3 days	Withdrawal	3
Maharani, B., et al. [17]	CR	1	Man/19	500 mg/day	Per os	Acute sinusitis	0,5 hour	Paracetamol, naphazoline	Acute anxiety with tactile hallucinations: blurring of vision, anxiety, confusion	No	No	1 hour	Withdrawal	3
Kang, G., et al. [18]	CR	1	Man/78	500 mg/day	IV	Pneumonia	2 days	Clopidogrel, choline alfoscerate, rebamipide, nizatidine, erdosteine	Confused mental state, difficulty of sleep initiation, and visual hallucinations	Diabetes mellitus, hypertension, old left basal ganglia hemorrhage, cerebellar lacunar infarct	No	1 day	Withdrawal, lamotrigine	3
Annadatha, A., et al. [19]	CR	1	Woman/62	500 mg/day	Per os	Acute bronchopneu monia	3 days	Paracetamol	Psychosis: auditory hallucination, irrelevant behavior	ND	No	48 hours	Withdrawal	3
Steuber, H., D. et al. [20]	CR	1	Woman/67	750 mg/day	Per os	Community acquired pneumonia	3 days	Azithromycin, ambrisentan, calcium citrate, fexofenadine, tramadol, cevimeline, tadalafil, vitamin E, fish oil, multivitamins	Psychosis: progressive altered mental status, intermittent repetitive statements and non-sensical statements	Pulmonary hypertension, rheumatoid arthritis, Sjogren's syndrome	Brain atrophy vs. hydrocephalus without changes in the last 3 years, one episode of steroid-induced psychosis	1 week	Withdrawal, Lorazepam, aripiprazole	3
Kogan, Y., et al. et al. [21]	CR	1	Woman/79	750 mg/day	Per os	Pneumonia	3 days	Ramipril, simvastatin	Acute delirium: confusion, irritability, disorientation, acoustic hallucinations,	Hypertension, hypercholesterolemia, allergy to penicillin	No	48 hours	Withdrawal	3

									impaired attention capacity					
Takser, L. and R. Grad, et al. [22]	CR	1	Woman/22	500 mg/day	Per os	Urinary tract infection	ND	ND	Acute psychosis: a sense of derealization, depersonalization, agitation, auditory hallucinations, insomnia, nightmares	ND	No	ND	ND	3
ŞAhan, E. and S. SetÇElİK et al. [23]	CR	1	Man/51	750 mg/day	Per os	Acute renal failure, pneumonia	48 h	Ceftriaxone	Delirium, visual hallucinations, suicidal attempt	No	No	24 hours	Withdrawal, amisulpride, haloperidol	3
Khan, A. and E. Khan et al. [24]	CR	1	Woman/66	ND	Per os	Severe urosepsis secondary to a urinary tract infection	1 day	Piperacillin, tazobactam	Delirium with psychotic features: disorientation, paranoid delusions, visual hallucinations, a bizarre and tangential thought process, physical agitation	Multiple sclerosis, Sjogren's syndrome	No	36 hours	Withdrawal	3
Husain, N.S.F., et al. [25]	CR	1	Woman/2	ND	ND	Preparation for bone marrow transplantation	Few hours after 1st dose	ANBL09P1 chemotherapeutic regimen, bisulfan, acyclovir, fluconazole, odansetron, lorazepam, aprepitant, cyproheptadine, cholecalciferol, omeprazole, ursodiol, oxycodone, morphine, melatonin	Delirium, sleep disturbance with episodes of agitation, disorientation, psychomotor agitation, aggression, mood lability, visual and tactile hallucinations	Stage IV neuroblastoma (MYCN nonamplified), 5 cycles of induction chemotherapy, episode of Klebsiella sepsis and 2 previous admissions for fever in the setting of neutropenia	No	ND	Withdrawal	3
Ghoshal, A., et al. [26]	CR	1	Man/17	500 mg/day	IV	Community-acquired pneumonia	10 minutes	Ceftriaxone, hydrocortisone, chlorpheneramine, adrenaline	Acute delirium: confused, disoriented, increased restlessness, agitation, violent behavior, visual hallucinations	Synovial sarcoma of right thigh with chest wall and lung metastasis	No	14 hours	Withdrawal, haloperidol midazolam	3
Chowdhry, V., et al. [27]	CR	1	Woman/ middle-aged	750 mg/day	Per os	Treated empirically - urinary tract infection	1 day	Midazolam, fentanyl, pancuronium, isoflurane, paracetamol	Delirium: anxiety, restlessness, insomnia, confusion, drowsiness, irritability, agitation	Cardiac surgery (mitral valve replacement)	No	72 hours	Withdrawal, clozapine	3
Agu, C., et al. [28]	CR	1	Man/25	ND	IV	Pneumonia	ND	Vancomycin, meropenem	Acute psychosis: horrid visual hallucination, agitation	ND	No	ND	Withdrawal	3
Singh, D., et al. [29]	CR	1	Man/28	500 mg/day	Per os	Pneumonia with urinary tract infection	3 days	Paracetamol, sodium citrate	Acute psychosis: restlessness, irritation and purposeless wandering, irrelevant and incoherent speech, insomnia, violent behavior,	No	No	2 days	Withdrawal, lorazepam	3

									auditory hallucinations, derangement of personality, loss of contact with reality					
Raj, V. and T.V. Murthy, et al. [30]	CR	1	Woman/13	500 mg/day	Per os	Acute bronchitis	2 hours	Antipyretics, antitussives	Delirium with psychotic features: restless with irrelevant speech, tremulousness, and a vibration like sensation in the body, confused, disoriented	No	No	4 days	Withdrawal	3
Lertxundi, U., et al. [31]	CR	1	Man/38	1st day: 1000 mg; next days: 500 mg/day	Per os	Pneumonia	3rd day	Escitalopram, valproic acid, pantoprazole, aripiprazole, clozapine, oseltamivir	Delirium, visual hallucinations	Hepatitis C positive, relapsing-remitting multiple sclerosis, multiple infra- and supratentorial lesions	Schizoaffective disorder	24 hours	Withdrawal	3
A. Pires, M., J. Mariz, et al. [32]	CR	1	Man/83	500 mg/day	ND	Urinary tract infection	24 hours	Furosemide, metolazone, warfarin, candesartan, spironolactone, theophylline, pravastatin, sitagliptin, glipizide, mianserin	Psychosis, delirium, agitation, bizarre behavior, incoherent dialog, aggressive, insomnia,	Atrial fibrillation, heart failure, hypertension, type 2 diabetes mellitus, urinary retention	No	48 hours	Withdrawal	3
Kocyigit, I., et al. [33]	CR	1	Man/55	500 mg/day	IV	Pneumonia	6th day	0,9% saline	Delirium with psychotic features: optic and acoustic hallucinations, hyperactivity, talking continuously	Renal dysfunction, essential hypertension, inguinal hernia surgery, tonsillectomy	No	48 hours	Withdrawal	3
Kandasamy, A. and D. Srinath, et al. [34]	CR	1	Man/30	500 mg/day	Per os	Lower respiratory tract illness	1st day	No	Insomnia, severe anxiety, irritable mood	ND	No	1 day	Withdrawal	3
	CR	1	Woman/30	500 mg/day	Per os	Lower respiratory tract illness	1st day	No	Insomnia, anxiety	ND	No	1 day	Withdrawal	
	CR	1	Man/32	750 mg/day	Per os	Lower respiratory tract illness	1st day	No	Insomnia, restlessness	ND	No	1 day	Withdrawal	
LaSalvia, E.A., G.J. Domek, and D.F. Gitlin et al. [35]	CR	1	Man/75	ND	ND	Postoperative high ostomy output and a wound infection	12h after 3rd dose	Ampicillin, metronidazole, trimethoprim-sulfamethoxazole, allopurinol, enalapril, propranolol	Anhedonia, hopelessness, poor concentration, psycho-motor retardation, and suicidal ideation	Colorectal cancer (surgical resection and chemotherapy)	No	4 days	Withdrawal, IV fluids	3
Slobodin, G., et al. [36]	CR	1	Man/83	500 mg/day	IV	Right lower lobe pneumonia	3rd day	Amoxicillin/clavulanate, clarithromycin	Delirium, visual hallucinations	Ischemic heart disease, coronary artery bypass surgery, mild renal failure, breast carcinoma	No	48 hours	Withdrawal	3

Moorthy, N., et al. [37]	CR	1	Man/50	500 mg/day	Per os	Persisting foot ulcer	3rd day	Amoxicillin, clavulanic acid, insulin, enalapril, atorvastatin	Acute psychosis: restlessness, irrelevant speech, abusive, violence, visual hallucinations, insomnia	Uncontrolled diabetes mellitus, hypertension, community acquired hospital pneumonia	No	48 hours	Withdrawal	3
Kiangkitiwan, B., et al. [38]	CR	1	Woman/42	500 mg/day	Per os	Sinusitis	2nd day	Meclizine, guaifenesin/ phenylpropanolamine	Acute delirium with psychosis: insomnia, decreased appetite, restlessness, agitation, paranoia, visual and auditory hallucinations	Sinus and urinary tract infections	Second-degree relative with bipolar disorder	24 hours	Withdrawal	3
Hakko, E., et al. [39]	CR	1	Man/73	500 mg/day	Per os	Community-acquired pneumonia	2 days	Ceftriaxone	Delirium: disorientation, confusion, impaired attention capacity, hyperactive, talking continuously	ND	ND	48 hours	Withdrawal	3
Yasuda, H., et al. [40]	CR	1	Man/67	300 mg/day	Per os	Upper respiratory infection	4th day	Fulfenamic acid	Visual hallucinations	Gastrectomy, cholecystectomy	Minor alcoholism, age-related brain atrophy	1 week	Withdrawal	3
**Ciprofloxacin**														
Stroud, S.G. et al. [81]	CR	1	Woman/56	500 mg/day	ND	Chronic osteomyelitis, postoperative	Next day	Vancomycin, ceftriaxone, ertapenem, antipsychotics, oxycodone	Acute delirium: paranoid delusions, audiovisual hallucinations, near-catatonia	Hypertension, chronic kidney disease, left open femur and tibial plateau fractures with draining wounds	No	2 days	Withdrawal (antipsychotics didn't help)	3
Song, G., et al. [41]	CR	1	Man/39	400 mg/day	IV	Severe pulmonary infection	2 days	Imipenem, vancomycin, meropenem, linezolid, caspofungin, piperacillin	Delirium: talkative, restless, and agitated, talking gibberish; suspected persecutory delusions, problems with sleeping	Aortic dissection, cardiac arrest, CPR, chronic hepatitis B, cerebrovascular disease	No	1 week	Withdrawal, rehydration therapy, vitamin B6, quetiapine	3
Nazef, C. and A. Arif et al. [42]	CR	1	Man/24	1000 mg/day	ND	Gastroenteritis, colitis	4 days	Metronidazole	Acute psychosis: auditory hallucinations, and suicidal ideations without intent or plan	No	Patient stated that he has had family members (paternal grandmother and uncle) with similar neurocognitive responses on ciprofloxacin therapy	2 days	Withdrawal	3
Weir, R.A., et al. [43]	CR	1	Woman/52	500 mg/day	Per os	Bacteremia	1 day	Cefepime	Profound psychosis and pseudobulbar affect: hallucinations, uncontrollable affect, paranoid delusions, vivid hallucinations, responding to internal	End-stage renal disease secondary to poorly controlled hypertension and chronic NSAIDs use	ND	4 days	Withdrawal, olanzapine	3

									stimuli, tremulousness, intact memory, attention, and orientation					
Rossi, G. and K. Mazoki et al. [44]	CR	1	Man/36	500 mg/day	Per os	Epididymitis	ND	ND	Acute paranoid psychosis: depression, anxiety, paranoia	No	Marijuana use	5 days	Withdrawal	3
Ransing, R.S. and D. Sarkar et al. [45]	CR	1	Woman/14	200 mg/day	IV	Acute gastroenteritis	2 days	IV fluids, antacids, probiotics	Mania: abnormal behavior, increased talkativeness, irritability, over demanding behavior, over familiarity, decreased need for sleep and elevated mood	ND	No	4 days	Lorazepam	3
Dikici, S., et al. [46]	CR	1	Woman/82	1000 mg/day	ND	Urinary tract infection	Next day	No	Acute delirium with psychotic features: shouting, falling asleep, abusive speech, confusion, hallucinations	Hypertension	ND	3 days	Withdrawal, quetiapine	3
Grimm, O. and B. Alm et al. [47]	CR	1	Woman/45	500 mg/day	Per os	Cystitis	7th day	ND	Acute polymorphic psychosis: reference and persecutory delusions with formal thought disorder, auditory hallucinations, emotional lability, aggressiveness	ND	ND	9 days	Aripiprazole	3
Dang, A., R. Kamat, and R.V. Padmanabh et al. [48]	CR	1	Woman/24	500 mg/day	Per os	Gastroenteritis	2 days	ND	Nightmares, fear, anxiousness, and nervousness, mood changes, irritable	ND	ND	1 day	Withdrawal	3
Jayathissa, S., et al. [49]	CR	1	Man/85	1000 mg/day	Per os	Infected right hip joint	7th day	ND	Delirium, hallucinations	ND	ND	ND	Withdrawal, clonazepam	3
Ahmed, A.I., et al. [50]	CR	1	Man/53	ND	ND	Recurrent prostatitis	3 weeks	Amlodipine (later changed into rampipril)	Suicide attempt	Hypertension, surgery for a cervical hernia	3 episodes of recurrent depression including with suicidal thoughts (each episode was preceded by 1-week treatment with ciprofloxacin); depressed mood for 1 year after the death of his wife (no	1 week	Withdrawal	3

											suicidal thoughts)			
Denysenko, L. and S.E. Nicolson et al. [51]	CR	1	Woman/60	400 mg/day	IV	Diverticular abscess	1 day	Metoclopramide, morphine, midazolam, fentanyl, cefoxitin, metronidazole, cinacalcet, lorazepam	Delirium, catatonia	Hypertension, chronic renal insufficiency	Cocaine abuse in remission for 4 years, one untreated 2-week episode of "depression" and "very little talking", delirium	5 days	Withdrawal, lorazepam	3
Labay-Kamara, U., S. Manning, and T. McMahon et Al. [52]	CR	1	Man/26	ND	ND	Colitis	2nd day	Levofloxacin, metronidazole	Serious suicide attempt	No	No	24 hours	Withdrawal, citalopram	3
Ben-Chetrit, E., N. Rothstein, and G. Munter et al. [53]	CR	1	Man/64	ND	ND	COPD with infected bronchiectasis	4th day	Cefuroxime, ramipril, ipratropium bromide inhaler	Acute psychosis: confusion, visual hallucinations	COPD, bronchiectasis, hypertension	ND	24 hours	Withdrawal	3
Ranjan, A. and S.K. et al. [54]	CR	1	Woman/22	200 mg/day (IV, 3 days)<br>500 mg/day (per os, 3 days)	IV, per os	Acute gastroenteritis	ND	"Supportive treatment"	Acute psychosis: fearfulness, psychomotor retardation, visual and auditory hallucinations, poor self-care	ND	No	2-3 days	Withdrawal	3
Dang, A., R. Kamat, and R.V et al. [55]	CR	1	Man/45	200 mg/day	ND	Urinary tract infection	Next day	No	Acute psychosis: delusional parasitosis, delusional beliefs, intensive visual hallucinations	Birth complications with asphyxia, cardiac surgery	An episode of depression after the divorce	2 days	Withdrawal	3
Bhalerao, S., et al. [56]	CR	1	Man/28	800 mg/day for 5 days; 1000 mg/day on the 6th day	IV	Postoperative after ECRP stenting	6th day	Sulfasalazine, metronidazole, cefazolin, gravol, ursodeoxycholic acid, folate, piperacillin-plus-tazobactam, thiamine, acyclovir	Delirium, mania episode	Primary sclerosing cholangitis, ulcerative colitis, ERCP stenting	No	15 days	Withdrawal, haloperidol, lorazepam, zopiclone, olanzapine	3
Norra, C., et al. [57]	CR	1	Woman/32	250 mg/day	Per os	Multidrug-resistant tuberculosis	2 days	Isoniazid, ethambutol, pyrazinamide, rifampicin	Psychotic symptoms, optic and acoustic hallucinations	No	A brother who had committed suicide, another brother treated for schizophrenia; patient was in mourning after	48 hours	Withdrawal, haloperidol	3

											brother's suicide			
Tripathi, A., S.I. Chen, and S. O'Sullivan et. Al. [58]	CR	1	Woman/27	1 drop/hour in each eye	Topic (eye drops)	Bacterial conjunctivitis	2 hours	No	Psychosis: visual and auditory hallucinations, disorganization of speech, behavioral disturbances	No	No	24-28 hours	Withdrawal	3
Al-Ghamdi, S.M. et. Al. [59]	CR	1	Woman/37	500 mg/day	ND	Urinary tract infection	1 day	Saline hydration, potassium chloride	Delirium: irritable, confused, not recognizing family members, abnormal behavior, disorientated to time, place and person	Chronic pyelonephritis and recurrent struvite stones	No	5 days	Withdrawal	3
Grassi, L., et al. [60]	CR	1	Woman/64	750 mg/day	Per os	COPD	3 days	Prednisone, digoxin, amiloride hydrochloride, hydrochlorothiazide, ranitidine, inhalatory fluticasone propionate	Delirium: depressed mood, reduced ability to concentrate, impairment in short-term memory, perception disturbances (misperceptions, illusions, and fragmented auditory hallucinations) and thought disorders (paranoid thinking)	Frequent relapses of COPD	No	3 days	Withdrawal, lorazepam	3
James, E.A. and A.Z. Demian et al. [61]	CR	1	Man/31	800 mg/day	ND	Intestinal sepsis (peritoneal soiling)	2 days	Piperacillin, gentamicin, vancomycin, metronidazole, fluconazole	Acute psychosis: paranoid delusions, visual hallucinations, behavioral disturbances	Gunshot in the stomach (underwent laparotomy)	No	9 days	Withdrawal	3
Jay, G.T. and J. Fitzgerald et al. [62]	CR	1	Woman/76	1000 mg/day	ND	Urosepsis (presumptive diagnosis)	36 hours	Oxybutynin, dicyclomine, imipramine, aspirin, betaxolol, pilocarpine eyedrops, baclofen, cholestyramine, ceftriaxone, gentamicin	Delirium	Paraplegic, chronic dermal ulcers, multiple rib/leg fractures, chronic indwelling catheter, osteomyelitis of the right foot, amputation above the right knee, colostomy, appendectomy	5 days prior to the admission hospitalization with fever, mental status changes and abdominal pain	3 days	Withdrawal, IV hydration	3
Mulhall, J.P. and L.S. Bergmann et al. [63]	CR	1	Woman/62	800 mg/ day, later switched to 1000 mg/day	IV, per os	Urinary tract infection	3 days	No	Acute psychosis: agitation, hallucinations, paranoid delusions	Anaphylactic reaction to penicillin in the past, recurrent urinary tract infections, pyolithotomy	No	36 hours	Withdrawal, haloperidol	3
Farrington, J., A. Stoudemire, and J. Tierney et al. [64]	CR	1	Woman/40	ND	ND	Bacterial meningitidis	4 days	Vancomycin, tobramycin, meperidine, oxycodone/acetamino phen, ceftazidime, droperidol, trimethoprim-	Delirium, agitation, visual hallucinations, generalized tremulousness	L5-S1 laminectomy, psuedomeningocele, tonsillectomy	No	3 days	Withdrawal, lorazepam,	3

								sulfamethoxazole, metoclopramide and amitriptyline, famotidine, chlordiazepoxide, diazepam						
Reeves, R.R. et al. [65]	CR	1	Man/49	500 mg/day	Per os	Prostatitis	2nd day	No	Psychosis: paranoid delusions	Peptic ulcer disease, recurrent back pain	The patient had some paranoic traits but did not fulfilled criteria for any disorder	3 days	Withdrawal, thiotixen	3
McCue, J.D. and J.R. Zandt et al. [66]	CR	1	Woman/76	750 mg/day	ND	Treated empirically because of a febrile	4th day	Carbidopa-levodopa, trimethoprim-sulfamethoxazole	Acute psychosis: confusion, hyperactivity, fearfulness, paranoid behavior	A right hemiparesis from a stroke	Parkinson's disease	4 days	Withdrawal, haloperidol	3
**Ofloxacin**														
Gautam, S.C., et al. [67]	CR	1	Woman/30	800 mg/day	ND	Urinary tract infection	2,5 day	Flavoxate hydrogen chloride	Psychosis: increased psychomotor activity, increased volume and rate of speech, irritable mood, irrelevant thoughts, delusions, auditory hallucinations	Pulmonary tuberculosis	No	24 hours	Withdrawal	3
Bhattacharya, A., et al. [68]	CR	1	Woman/4	140 mg/day IV for 2-3 days; 800 mg/day per os the next day	IV, per os	Acute gastroenteritis	1 day after the dose of 800 mg	Ondansetron, IV fluids	Delirium, bimodal hallucinatory psychosis: tactile and visual hallucinations	No	No	72 hours	Withdrawal, olanzapine	3
Chauhan, U., et al. [69]	CR	1	Woman/5	100 mg/day	Per os	Gastroenteritis	3 days	Sodium valproate, lamotrigine, ornidazole, ondansetron	Visual hallucinations	Seizure disorder (seizure-free for the past 1 year)	ND	24 hours	Withdrawal	3
Koul, S., et al. et al. [70]	CR	1	Woman/18	800 mg/day	ND	Urinary tract infection	2 days	Co-amoxiclav, metronidazole	Psychosis: paranoid delusions, delusions of reference and control, third person auditory hallucinations, suicidal ideation, tearfulness, psychomotor retardation, psychomotor agitation	ND	No	4 days	Withdrawal	3
Guven, E.O., et al. [71]	CR	1	Man/75	400 mg/day	ND	Postoperative after trans vesical prostatectomy	24-48 hours	No	Psychosis: disorientation, hallucination, disorientation, echolalia, echopraxia, swearing, hypersalivation,	ND	ND	48 hours	Withdrawal	3

									inability to swallow, cogwheel rigidity, blurring of consciousness, hyper irritative answer to vocal stimulation					
	CR	1	Woman/50	400 mg/day	IV	Acute pyelonephritis	3rd day	“Supportive therapy”	Psychosis: disorientation, hallucinations	ND	ND	24 hours	Withdrawal	3
Hall, C.E., et al. [72]	CR	1	Woman/32	800 mg/day	ND	Pelvic inflammatory disease	A few hours after 1st dose	Doxycycline, metronidazole,	Psychosis; nihilistic delusions, psychotic anxiety, panic attack	Gastro-esophageal reflux disease, irritable bowel syndrome	A moderate depressive disorder, panic disorder requiring antidepressant therapy	48 hours	Withdrawal	3
**Moxifloxacin**														
Gautam, S.C., et al. [67]	CR	1	Woman/30	800 mg/day	ND	Urinary tract infection	2,5 day	Flavoxate hydrogen chloride	Psychosis: increased psychomotor activity, increased volume and rate of speech, irritable mood, irrelevant thoughts, delusions, auditory hallucinations	Pulmonary tuberculosis	No	24 hours	Withdrawal	3
Bhattacharya, A., R. et al. [68]	CR	1	Woman/4	140 mg/day IV for 2-3 days; 800 mg/day per os the next day	IV, per os	Acute gastroenteritis	1 day after the dose of 800 mg	Ondansetron, IV fluids	Delirium, bimodal hallucinatory psychosis: tactile and visual hallucinations	No	No	72 hours	Withdrawal, olanzapine	3
Chauhan, U., P. et al. [69]	CR	1	Woman/5	100 mg/day	Per os	Gastroenteritis	3 days	Sodium valproate, lamotrigine, ornidazole, ondansetron	Visual hallucinations	Seizure disorder (seizure-free for the past 1 year)	ND	24 hours	Withdrawal	3
Koul, S., et al. [70]	CR	1	Woman/18	800 mg/day	ND	Urinary tract infection	2 days	Co-amoxiclav, metronidazole	Psychosis: paranoid delusions, delusions of reference and control, third person auditory hallucinations, suicidal ideation, tearfulness, psychomotor retardation, psychomotor agitation	ND	No	4 days	Withdrawal	3
Guven, E.O., et al. [71]	CR	1	Man/75	400 mg/day	ND	Postoperative after trans vesical prostatectomy	24-48 hours	No	Psychosis: disorientation, hallucination, disorientation, echolalia, echopraxia, swearing, hypersalivation, inability to swallow,	ND	ND	48 hours	Withdrawal	3

									cogwheel rigidity, blurring of consciousness, hyper irritative answer to vocal stimulation					
	CR	1	Woman/50	400 mg/day	IV	Acute pyelonephritis	3rd day	"Supportive therapy"	Psychosis: disorientation, hallucinations	ND	ND	24 hours	Withdrawal	3
Hall, C.E., et al. [72]	CR	1	Woman/32	800 mg/day	ND	Pelvic inflammatory disease	A few hours after 1st dose	Doxycycline, metronidazole,	Psychosis; nihilistic delusions, psychotic anxiety, panic attack	Gastro-esophageal reflux disease, irritable bowel syndrome	A moderate depressive disorder, panic disorder requiring antidepressant therapy	48 hours	Withdrawal	3
Moxifloxacin														
Uz, B., et al. [73]	CR	1	Woman/66	400 mg/day	Per os	Left lobar pneumonia	6,5 hours	Erdosteine, paracetamol, amlodipine, acetylsalicylic acid	Complex visual hallucinations, logorrhea, euphoria, behavioral changes	Essential hypertension, osteoporosis	No	36 hours	Withdrawal	3
Higdon, E., et al. [74]	CR	1	Woman/24	400 mg/day	IV	Severe sepsis secondary to community-acquired pneumococcal pneumonia	2 days	Hydroxy- chloroquine, prednisone, vancomycin, piperacillin/tazobactam, ciprofloxacin, ceftriaxone	Visual hallucinations	Systemic lupus erythematosus	No	1 day	Withdrawal	3
Mazhar, F., et al. [75]	CR	1	Man/40	400 mg/day	Per os	Acute bronchitis	72 hours	Paracetamol	Acute psychosis: persistent insomnia and impaired visual and auditory perception, awareness of unreality	No	No	12 hours	Withdrawal	3
Karagoz, E., et al., [76]	CR	1	Woman/60	400 mg/day	IV	Atypical pneumonia	1 hour after 2nd dose	Amoxicillin-clavulanate	Acute delirium: visual hallucinations, apathic behaviors, inability to remember her family members, irrelevant talk, pressured speech, loose associations	ND	ND	3 days	Withdrawal, Quetiapine	3
Pepele, M.S., et al. [77]	CR	1	Woman/65	400 mg/day	Per os	COPD	2 hours	Acetylcysteine	Visual hallucinations	COPD	No	6 hours	Withdrawal	3
Tasleem, H. et al. [78]	CR	1	Woman/70	400 mg/day	IV, per os	Streptococcus pneumonia, bacteremia, urinary tract infection, colitis	7th day of the treatment; the same day as oral dose	Prednisone, hydralazine, clonidine, metoprolol, zolpidem, levothyroxine, furosemide, cyclosporine, levofloxacin, metronidazole, filgrastim, vancomycin	Delirium with visual hallucinations	Hypertension, coronary artery disease, renal transplantation, COPD, hypothyroidism, excision of squamous cell carcinoma on both shins	Daily marijuana use	1 day	Withdrawal	3
**Gemifloxacin**														

Sharma, D.D., et al. [79]	CR	1	Woman/36	320 mg/day	ND	Upper respiratory tract infection	1st day	Loratadine	Acute dystonia, difficulty in sleeping, restlessness	No	No	5-10 minutes after promethazine administratio n	Withdrawal, promethazine	3
Barrett, M.J. et al. [80]	CR	1	Woman/67	320 mg/day	Per os	Upper respiratory tract infection (suspected)	0,5 hour – 1 day	No	Acute alteration in mental status: dysphasic, unable to follow commands, agitated	No	No	2 days	Withdrawal	3
